# Comparative study of the adsorption of acetaminophen on activated carbons in simulated gastric fluid

**DOI:** 10.1186/2193-1801-3-48

**Published:** 2014-01-24

**Authors:** Carlos A Rey-Mafull, Juan E Tacoronte, Raquel Garcia, Jorge Tobella, Julio C Llópiz, Alberto Iglesias, Dachamir Hotza

**Affiliations:** Instituto de Ciencia y Tecnología de Materiales, Facultad de Química, Universidad de la Habana, Havana, Cuba; Centro de Ingeniería e Investigaciones Químicas, Havana, Cuba; Laboratorio Reinaldo Gutiérrez, Havana, Cuba; Laboratorios MEDSOL, Havana, Cuba; Facultad de Física, Universidad de la Habana, Havana, Cuba; Programa de Pós-Graduação em Ciência e Engenharia de Materiais (PGMAT), Universidade Federal de Santa Catarina (UFSC), Florianópolis, Brazil

**Keywords:** Activated carbon, Acetaminophen, Adsorption, Isotherms, Simulated gastric fluid

## Abstract

Samples of commercial activated carbons (AC) obtained from different sources: Norit E Supra USP, Norit B Test EUR, and ML (Baracoa, Cuba) were investigated. The adsorption of acetaminophen, C_o_ = 2500 mg/L, occured in simulated gastric fluid (SGF) at pH 1.2 in contact with activated carbon for 4 h at 310 K in water bath with stirring. Residual acetaminophen was monitored by UV visible. The results were converted to scale adsorption isotherms using alternative models: Langmuir TI and TII, Freundlich, Dubinin-Radushkevich (DR) and Temkin. Linearized forms of the characteristic parameters were obtained in each case. The models that best fit the experimental data were Langmuir TI and Temkin with R^2^ ≥0.98. The regression best fits followed the sequence: Langmuir TI = Temkin > DR > LangmuirTII > Freundlich. The microporosity determined by adsorption of CO_2_ at 273 K with a single term DR regression presented R^2^ > 0.98. The adsorption of acetaminophen may occur in specific sites and also in the basal region. It was determined that the adsorption process of acetaminophen on AC in SGF is spontaneous (ΔG <0) and exothermic (−ΔH_ads._). Moreover, the area occupied by the acetaminophen molecule was calculated with a relative error from 7.8 to 50%.

## Introduction

Activated carbon (AC) can be applied orally as an antidote to different intoxications. Several studies, both in vitro and in vivo have demonstrated the capacity of activated carbon to adsorb numerous toxic compounds (Neuvonen & Olkkola 1989; Alaspaa et al. 2000; Ho et al. 1989; Pond 1986; McGoodwin & Schaeffer 2000; Cooper et al. 2005; Hoegberg et al. 2003; Modi et al. 1994; Hoegberg et al. 2002; El-Kemary et al. 2011). The 1-15acetaminophen (*N-*acetyl- *p-*aminophenol) is a drug with analgesic properties, without clinically significant anti-inflammatory properties. It acts by inhibiting prostaglandin synthesis, cellular mediators responsible for the onset of pain. It also has antipyretic effects. It is available usually in the form of capsules, tablets, suppositories, and drops for oral administration. It is a common ingredient in a variety of products against cold and flu.

Its low price and widespread availability have resulted in frequent cases of overdose. In the indicated doses, acetaminophen presents no effect on the gastric mucosa, blood clotting or kidneys, but the liver might be severely affected.

Adsorption capacity of AC depends on the nature of the adsorbent (pore structure, functional groups, ash content) as well as the nature of the adsorbate (functional groups, polarity, molecular size and weight). The type of precursor and the process of activation determine basic properties of AC such as surface area and pore size distribution. The ACs has strong heterogeneous surfaces, in both geometrical and chemical character. The geometrical heterogeneity is the result of differences in the size and shape of pores as well as pits, and vacancies. Chemical heterogeneity is associated to different functional groups at a surface (mainly oxygen) and to various surface contaminants. Both heterogeneities contribute to unique adsorption properties of activated carbons (Neuvonen & Olkkola 1989; Alaspaa et al. 2000; Ho et al. 1989; Pond 1986; McGoodwin & Schaeffer 2000; Cooper et al. 2005; Hoegberg et al. 2003; Modi et al. 1994; Hoegberg et al. 2002; El-Kemary et al. 2011; American Academy of Clinical Toxicology (AACT); European Association of Poisons Centres and Clinical Toxicologists (EAPCCT) 1999; Bryant et al. 2003; Neuvonen et al. 1984; Neuvonen 1982; Neuvonen & Olkkola 1984; Yamamoto et al. 2007). The purpose of this study is to investigate the efficacy of AC to remove acetaminophen dissolved in simulated gastric fluid (SGF).

## Materials and methods

### Acetaminophen

**Figure 1:**
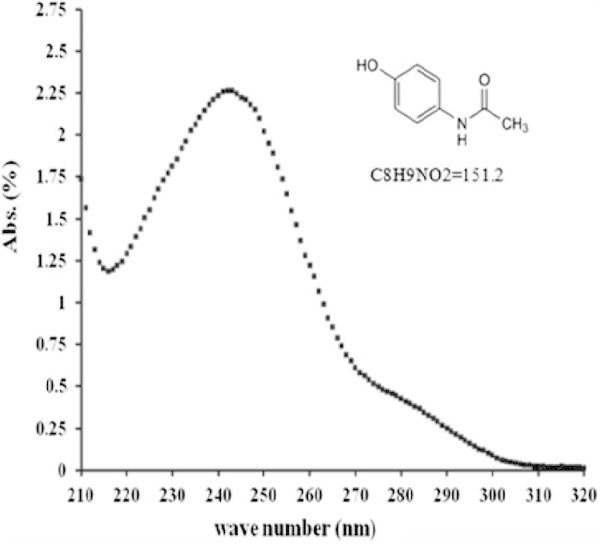
**UV**-**visible spectrum of acetaminophen in SGF at pH 1.2.**

Acetaminophen (> 99%) has been purchased from Sigma-Aldrich. It is a weak acid (pK_a_ = 9.5) presenting an aromatic hydroxyl group, and solubility in water at 20°C of 1.4 g/100 mL. The Food and Drug Administration (FDA) suggests spectrophotometry UV in aqueous acid solution, λ_max_ = 245 nm, as reference method for its quantification. The UV-visible spectrum is showed in Figure 1 (El-Kemary et al. 2011; Gyamlani & Parikh 2002).

### Activated carbon

Norit B (NB) Test EUR (Germany) and Norit E (NE) Supra USP (Holland) were taken as references. The activated carbon ML was supplied by the Baracoa Activated Carbon Plant (Cuba) and purified by acid/basic treatment (Rey-Mafull et al. 2007; Rey-Mafull et al. 2010). The particle sizes correspond to 100% < 250 μm. All carbons follow the requirements of the standard actived charcoal according to the United States Pharmacopeia (USP30-NF25, 2007).

### Simulated gastric fluid (SGF)

The SGF was prepared according to USP 30 as follows: 2 g NaCl were dissolved in 7 mL of concentrated HCl and filled up to 1 L with distilled water free of CO_2_ and simultaneously adjusting the pH of the solution to 1.2. Acetaminophen was added to the SGF solution reaching a concentration 2500 mg/L. The calibration curve of acetaminophen in SGF was performed using a UV/VIS spectrophotometer (Ultrospec 2100 pro from Amersham Biosciences). The optical density of all samples was determined with maximum absorbance at λ_max_ = 245 nm in the zone of Lambert Beer transmittance. The calibration curve was adjusted using the linear or quadratic regression analysis. Each experiment was performed by triplicate.

### Batch equilibrium experiments and analytical method

Stock solution of acetaminophen at the concentration of 2500 mg/L was prepared by dissolving acetaminophen in 1 L of SGF readjusted to pH 1.2. Analyses were repeated in triplicate and averaged 15 experimental runs were conducted for each type of AC. A particle size distribution analysis was performed for the AC samples. During adsorption the amount of carbon varied in the range of 0.001 to 0.27 g. AC samples were added to the solution of acetaminophen dissolved in SFG and kept under constant stirring at100 rpm for 4 h at room temperature (37.0 ± 0.1°C). The samples were then filtered and the 5 ml solution liquid extract was taken for the UV/VIS analysis. The amount of acetaminophen adsorbed by the activated carbon was calculated by mass balance. Previously established linear Beer-Lambert relationships were used in the concentration analysis. For the solutions with higher concentrations, dilution was required to operate the analysis in the Beer-Lambert region. Absorbance readings were taken from the calibration curve which determines the equilibrium concentration corresponding to each of the points of the isotherm. The amount of adsorption at equilibrium, *q*_e_ (mg/g), was calculated by Eq. 1.1

where *C*_*0*_*(mg/mL)* is the initial concentration (*t* = 0), *C*_*e*_*(mg/ mL)* is the equilibrium concentration (*t = 4 h*), *M* is the mass of carbon *(g)* and *V* is the volume of the solution *(L).*

### Adsorption isotherms CO_2_ at 273 K

The microposity of the carbons was characterized by using CO_2_ adsorption isotherms measured at 273 K (ASAP 2050 V1.00 E System from Micromeritics). Prior to experiments the samples were outgassed at 240°C during 24 h. The adsorption isotherms parameters were calculated by applying the Dubinin-Radushkevich (DR) equation (Eq. 2) (Smisek & Cerny 1970; Bradley & Rand 1995; McEnaney 1987; Stoeckli 1998; Dubinin & Stoeckli 1980; Stoeckli et al. 2001):2

where *W (cm*^*3*^*/g)* represents the volume filled at temperature *T (K)*, *β* is the similarity coefficient, *E*_*0*_*(kJ/mol)* characteristic energy and *W*_*0*_*(cm*^*3*^*/g)* is the maximum adsorption capacity that is related with micropores volume and *A* is the differential molar work or the change in Gibbs free energy ΔG, defined by Eq. 33

where *P*_*0*_*/P* relative pressure, *R* is the ideal gas constant (0.00831 kJ/mol K) and *T* is the temperature (K). The volume of micropores was calculated by the following expression (Eq. 4):4

where *V*_*m*_ represents the CO_2_ molar volume at 273 K. The so-called characteristic energy *E*_*0*_ is related to the average micropore width, *W*_*m*_ (nm), when a pore size lies between 0.45 to 2.5 nm, by the following expression (Eq. 5) (Stoeckli 1998; Dubinin & Stoeckli 1980; Stoeckli et al. 2001).5

The average gyration radius, *R*_*g*_(nm), is determined by using the Dubinin–Steockli relationship (Eq. 6):6

### Adsorption isotherms

An equilibrium isotherm expresses the relationship between the amounts of adsorbate removed from solution at equilibrium by unit of mass of adsorbent at constant temperature. In this study, equilibrium data of the acetaminophen adsorption was processed by alternative two-parameter isotherms including: Langmuir (TI and TII), Freundlich, Dubinin–Radushkevich (DR) and Temkin. The linear expressions of those isotherm equations and the way to obtain the isotherm parameters are given in Table 1. The method of least squares was used for obtaining the trend lines. The characteristic parameters were determined from the respective linear form (Quesada et al. 2009; Passé et al. 2009; Behnamfard & Salarirad 2009; Richard et al. 2009; Xin et al. 2011; Ahmad & Rahman 2011)*.*

**Table 1:** **Isotherms and their linearized expressions**

Isotherms	Expressions	Linear expressions	Plots
Langmuir			
	ΔG = − RTln [K_L_]		
Freundlich		lnq_e_ = lnK_F_ + n^− 1^lnC_e_	lnq_e_vslnC_e_
Dubinin-Radushkevih (DR**)**		lnq_e_ = lnq_max_ − Dϵ^2^	lnq_e_vs*ϵ* ^2^

	*E*_0_ = [2D]^− 0.5^		
Temkin		q_e_ = BlnK_TK_ + BlnC_e_	q_e_vslnC_e_
			

### Functional groups identification (FTIR)

FTIR spectra for different activated carbon samples (4000–400 cm^-1^) were recorded on a FTIR spectrophotometer (Nicolet 50X), using KBr pellets containing 0.1 wt% carbon. Those pellets were dried for 8 h at 100°C before the spectra were recorded.

## Results and discussion

### Textural properties of the carbons

The results obtained by applying the DR model to the CO_2_ isotherm are summarized in Table 2. The R^2^ obtained for the DR isotherms were above 0.98. The activated carbons NE and ML presented very similar microporosities.

**Table 2:** **Textural characterization by adsorption of CO**_**2**_ **at 273 K**

Adsorbent	***W0 (cm3/g)***	***E0 (kJ/mol)***	***Rg*** (nm)	***Wm*** (nm)	***Vmicro*** (cm3/g)	R^2^
Norit E Supra USP (NE)	5.64	6.17	1.61	3.11	0.31	0.98
Norit B Test EUR (NB)	14.15	8.28	1.37	2.64	0.68	0.99
Baracoa, Cuba (ML)	5.65	7.87	1.44	2.78	0.39	0.99

### Identification of surface functional groups

**Figure 2:**
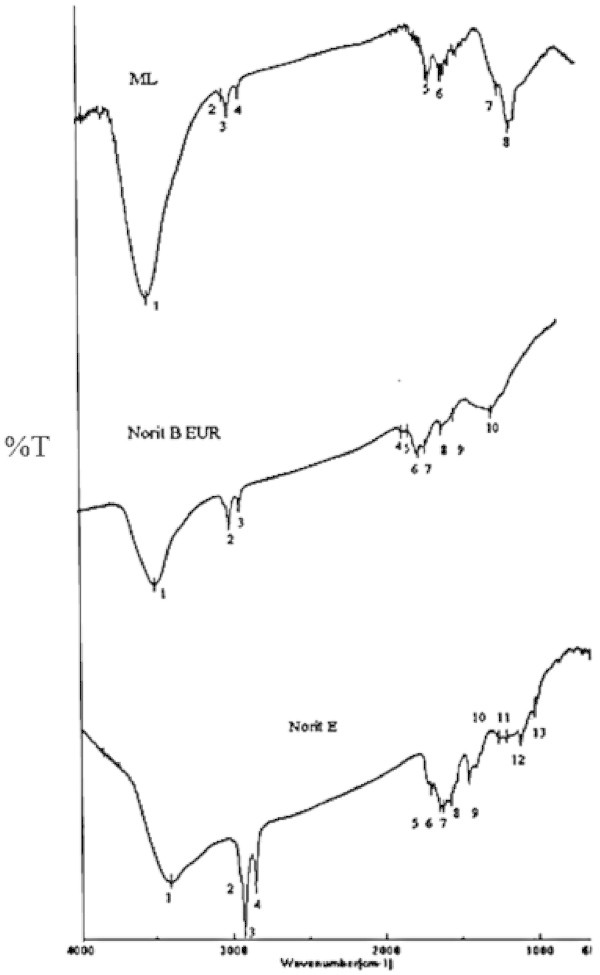
**FTIR spectra for activated carbons.**

Common bands were identified for the three activated carbons (3424, 2852, 2921 and 1125 cm^-1^), Figure 2. The band in 3424 cm^-1^ is assigned to carbonyl group -OH stretching. The peaks at 2852 and 2921 cm^–1^ are due to the presence of aliphatic CH, CH_2,_ and CH_3_ groups, and the one at1125 cm^-1^ is expected to be related to carboxylic –OH group. The sample NB shows the band 1737 cm^-1^ which can be assigned to lactone group, whereas in sample NE a peak is observed at 1710 cm^-1^ which can be assigned either to lactone or to nonaromatic carboxyl groups, for which the C = O stretching has been reported to occur at 1712 cm^-1^. The bands from 1600 to 1650 cm^-1^ can be assigned to C = O quinonics groups. In those ranges the following bands were found: 1652 cm^-1^ for NE, 1629 cm^-1^ for NB/NE, and 1641 cm^-1^ for ML. The band 1578 cm^-1^, which is common to NB/NE, has not been interpreted unequivocally. This has been assigned to aromatic ring stretching couplet of highly conjugated carbonyl groups. The peak at 2900 cm^-1^ corresponds to the following functional groups: C-H, -CH_2_, - CH_3_. Moreover, a series of peaks of moderate intensity between wave numbers 1400 and 1700 cm^-1^ can be attributed to the elongation of the functional groups C = O and C = C due to the presence of ketones, esters, aldehydes and carboxylic acids. At 1038 cm^-1^ NE presents a peak which corresponds to alcoholic C-O vibration stretching (Moreno et al. 2000; Moreno 2004; Pradhan & Sandle 1999; Terzyk et al. 2003; Terzyk 2002; Liu et al. 2009; Liu et al. 2010).

### Equilibrium isotherms

**Figure 3:**
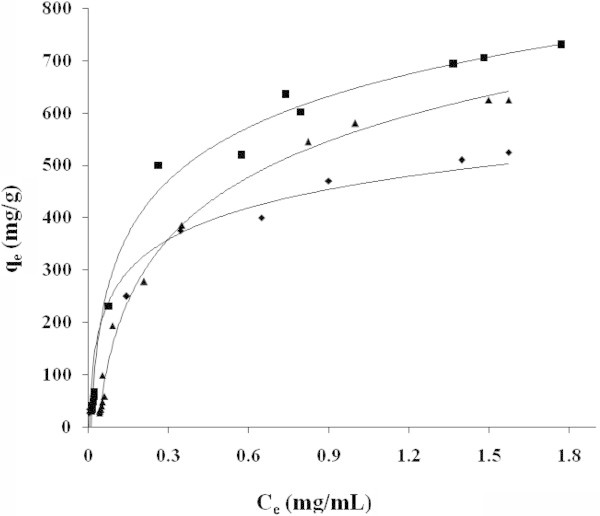
**Experimental data of adsorption isotherms profile of acetaminophen in SGF, NB (■) NE (♦) ML (▲).**

The experimental adsorption data of acetaminophen on NE, NB and ML activated carbons at 37°C are plotted in Figure 3. The isotherms show that NB has the highest adsorption capacity and the highest *V*_*mico*_ (cm^3^/g), but there is no linear relationship between micropores and the adsorption capacity, because ML presented similar values at a lower *Vmicro* (Tables 2, 3 and 4). This result illustrates that no simple relationship exists between the adsorption capacity of carbons and their textural properties. This has also been reported by (Moreno et al. 2000; Moreno 2004), who have shown that the surface chemistry of the carbon has to be considered an important factor in the adsorption mechanism in diluted aqueous solutions.

**Table 3:** **Characteristic parameters of models**

Activated carbon	Langmuir				Freundlich		Dubinin-Radushkevich		Temkin	
	TI		TII							
	Parameters	R^2^	Parameters	R^2^	Parameters	R^2^	Parameters	R^2^	Parameters	R^2^
	q_m_ = 840	0.99	q_m_ = 1315.8	0.99	K_F_ = 685	0.96	q_max_ = 685	0.99	b = 17.5	0.99
NB	K_LI_ = 3.6		K_LII_ = 2		n = 1.6		D = 0.018		K_TK_ = 82	
	q_m_ = 555	0.99	q_m_ = 356	0.89	K_F_ = 503	0.98	q_max_ = 458	0.98	b = 29.6	0.98
NE	K_LI_ = 6		K_LII_ = 19		n = 1.9		D = 0.016		K_TK_ = 202	
	q_m_ = 769	0.99	q_m_ = 1462	0.95	K_F_ = 605	0.88	q_max_ = 632	0.96	b = 7.2	0.99
ML	K_LI_ = 3.3		K_LII_ = 1		n = 1.7		D = 0.04		K_TK_ = 27	

**Table 4:** **Free energy change Gibbs** ***(ΔG)*** **calculated by the Langmuir and Temkin equations and characteristic energy** ***(E)*** **calculated by the Dubinin Radushkevich’s equation**

Parameters	Models	NE	NB	ML
ΔG (kJ/mol)	Langmuir (TI)	− 4.6	− 3.3	− 3.25
ΔG (kJ/mol)	Temkin	−13.7	−11.4	−8.5
E (kJ/mol)	Dubinin-Radushkevich	5	5	4.8

Inumerous works have been conducted in order to elucidate the mechanism of adsorption of many molecules on different adsorbents (Passé et al. 2009; Behnamfard & Salarirad 2009; Richard et al. 2009; Xin et al. 2011; Ahmad & Rahman 2011; Moreno et al. 2000; Moreno 2004; Pradhan & Sandle 1999; Terzyk et al. 2003). Those publications reveal that adsorption of organic molecules from dilute aqueous solutions on carbon-based materials is a complex interaction between electrostatic and non-electrostatic forces. Moreover, both interactions depend on the characteristics of the adsorbent and adsorbate, as well as on chemical properties of the solution. It was observed that the adsorption capacity is negatively influenced by the presence of basic surface groups. Terzyk et al. 2003 reported a decrease of acetaminophen maximal adsorption capacity as the total amount of surface basic groups and carbonyls increases on ACs. It was postulated that the acetaminophen molecule interacts by the OH^-^ group with carbon basic surfaces, and the repulsion effect occurs between the CO group of this molecule and similar groups attached to the surface (Yamamoto et al. 2007; Terzyk et al. 2003; Terzyk 2002).

**Figure 4:**
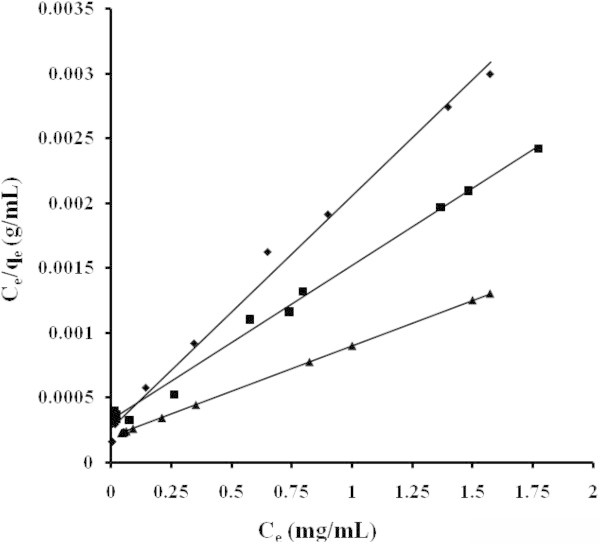
**Adsorption isotherms adjusted in Langmuir’s TI coordinate NB (■) NE (♦) ML (▲).**

**Figure 5:**
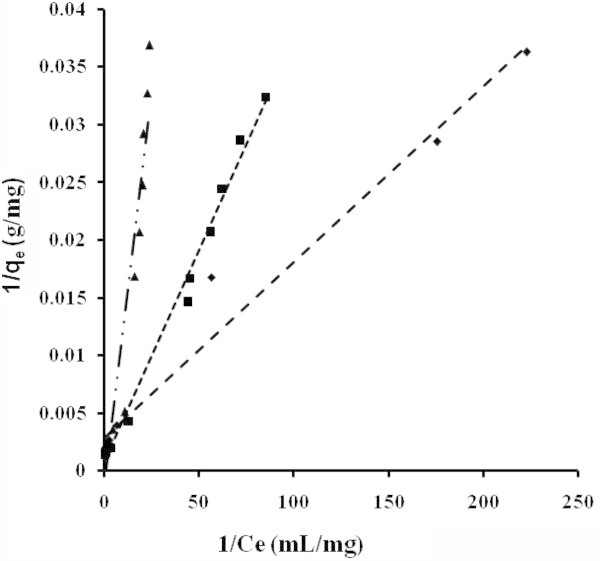
**Adsorption isotherms adjusted in Langmuir’s TII coordinate NB (■) NE (♦) ML (▲).**

**Figure 6:**
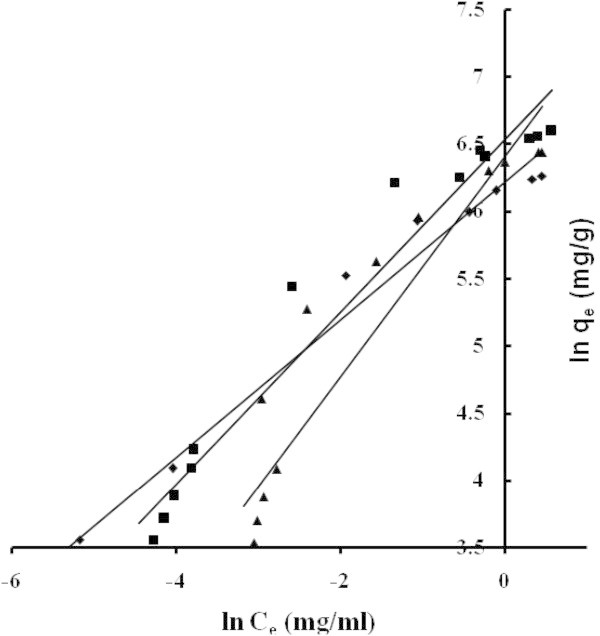
**Adsorption isotherms adjusted in Freundlich’s coordinate NB (■) NE (♦) ML (▲).**

**Figure 7:**
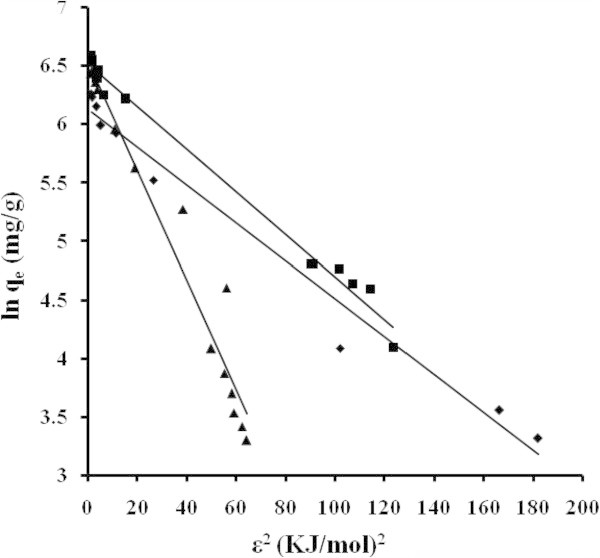
**Adsorption isotherms adjusted in** **Dubinin-Radushkevich`s** **coordinate NB (■) NE (♦) ML (▲).**

**Figure 8:**
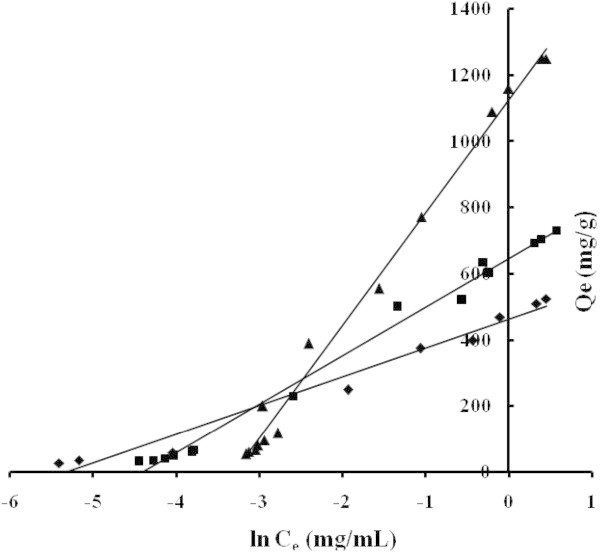
**Adsorption isotherms adjusted in Temkin’s coordinate NB (■) NE (♦) ML (▲).**

The applicability of the isotherm equation to describe the adsorption process was evaluated by the correlation coefficients, *R*^2^. The relative parameters of five different linearized forms of isotherms were obtained from the plots as shown in Figures 4, 5, 6, 7 and 8 and Tables 3 to 4. The adsorption isotherm models fitted the data in the following order: *LTI = LTII = T > F = DR* (NB), *LTI > F > T = DR* (NE) and *T > LTI > DR* (ML).

The Langmuir adsorption model describes monolayer adsorption of adsorbate onto a homogeneous adsorbent surface. Moreover, there is negligible interaction between the adsorbed molecules and adsorption sites having uniform energies. The Langmuir isotherm accounts for surface-coverage by balancing the relative rates of uptake and release, the former being proportional to the fraction of the surface is open, while the latter is proportional to the fraction that is covered. The equilibrium constant for those rates is *K* (L/mg), which also corresponds to the Henry’s law coefficient. When the fluid concentration is very high, a monolayer forms on the adsorbent surface, having a loading of *q*_*max*_*.*(mg/g).

The essential characteristics of Langmuir equation can be expressed in terms of dimensionless separation factor, Eq. 7.7

The term *R*_*L*_ indicates the shape of the isotherm as follows. When the parameters, *R*_*L*_ >1 (unfavorable isotherm), *R*_*L*_ = 1 (linear isotherm), 0 < *R*_*L*_ < 1 (favorable isotherm). In all cases *R*_*L*_*(3.99 × 10*^*-4*^*)* expresses that the isotherm has a favorable behavior.

The Dubinin- Radushkevich equation: assumes that the amount adsorbed corresponding to any adsorbate concentration is a Gaussian function of the Polanyi potential The development of this theory is based on the concept of the characteristic curve and Polanyi adsorption potential (∂ E/∂ T = 0) is applied to describe the adsorption in micropores. A linear plot of ln *q*_*e*_ against ϵ^*2*^ (kJ/mol)^2^ would give the value of *q*_*max*_ (mg/g) and *D* (mol^2^kJ^-2^), from the intercept and slope. The calculated value of *E* (kJ/mol) is shown in the Tables 3 to 4.

**Figure 9:**
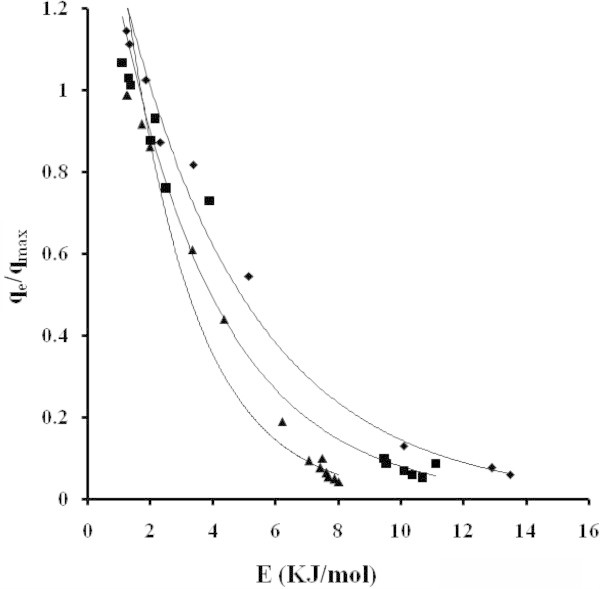
**Characteristic curves for the adsorption of acetaminophen dissolved in SGF (pH 1.2, T = 310 K) NB (■) NE (♦) ML (▲).**

From the Langmuir and DR equations, spontaneity of the systems and the energy involved during the adsorption process was estimated. Other values, related with this system, were calculated and reported Terzyk (2002), as enthalpy of immersion (16, 11, 8 and 6 kJ/mol). The magnitude of the ΔH values lies in the range of 2.1 to 20.9 and 80 to 200 kJ/mol for physical and chemical adsorption, respectively. Generally, the ΔG is in the range of 0 to −20 kJ/mol and −80 to −400 kJ/mol for physical and chemical adsorption (Liu et al. 2009; Liu et al. 2010; Li et al. 2005). A characteristic curve of acetaminophen for SGF obtained from the DR equation, Figure 9, shows that the values of energy in the environment previously predicted. To the extent that the degree of coverage decreases to 0.1, the values of the energies can be found between 7.5 to 12.5 kJ/mol. They reach the average value of 5 kJ/mol for coating q_e_/q_max_ = 0.4 to 0.5.

Terzyk et al. (2012) proposed the molecular cross sectional area (m.c.s.a) of acetaminophen as 60.2 Å^2^. In Table 5 the values estimated of the molecular cross sectional area of acetaminophen by Langmuir, DR and Freundlich equations are presented. To calculate the molecular cross sectional area of acetaminophen (Å^2^) the following Eq. 8 was employed:8

where *A*_*sp*_ (m^2^/g) is the specific surface area of the activated carbon, obtained from BET analysis of N_2_ vapor adsorption data, *MW* is the molecular weight of acetaminophen, *K* is the number of moles of acetaminophen adsorbed per gram of adsorbent at maximum surface coverage, and *N*_*0*_ is the Avogadro’s number (Wurster & Aburub 2006; Wurster et al. 2003).

**Table 5:** **Estimated calculation of the value of specific surface area for acetaminophen molecule taking as reference the value 60.2 Å** ^**2**^ **proposed by** Terzyk et al. (2012)

	Langmuir		Dubinin-Radushkevich		Freundlich	
Adsorbent	m.c.s.a (Å^2^)	Relative error (%)	m.c.s.a (Å^2^)	Relative error (%)	m.c.s.a (Å^2^)	Relative error (%)
NB	42.7	20	55.5	7.8	52	14
NE	39.3	35	47.6	21	43.4	28
ML	23.5	62	28.3	50	29.8	50

The effectiveness of the degree of compaction of the molecule of acetaminophen on the surface of the activated carbon is related to the optimal distribution of the sites of adsorption to the maximum extent of packaging of this molecule. The determination of the area of the molecule of acetaminophen , in their non-ionized state, in SGF shows a better orientation on the surface of activated carbon, in an order of priority NB > NE > ML. The cause could be a better distribution of the active sites of adsorption, in both textural and functional plane respectively. However, with regard to the relative error show models in the estimated calculation, the order is as follows: Langmuir > Freundlich > DR. But in all cases the relative error is greater than 10%.

The Temkin model considered the effects of some indirect adsorbent/adsorbate interactions on adsorption isotherms. As a result of adsorbent/adsorbate interactions, the heat of adsorption of all the molecules in the layer would decrease linearly with coverage. This is a correction of Langmuir equation, and introduces the influence of temperature on the adsorption. In this case, b (J/mol) is the Temkim constant related to heat of sorption and K_TK_ is the Temkin isotherm constant (L/g). The variation of adsorption energy b is positive for all the studied AC, Table 3, which indicates that the adsorption reaction is exothermic (−ΔH_ads_) (Hameed et al. 2008).

The empirical Freundlich model is based upon the assumption of multilayer formation of adsorbate on the heterogeneous solid surface of the adsorbent and assumes that the stronger binding sites are occupied first and that the binding strength decreases with the increasing degree of site occupation. The values *Q*_*max*_ and *1/n* are Freundlich constants related to adsorption capacity and intensity of adsorption, respectively. The lower fractional values of 1/*n* [0 < (*1/n*) <1] indicate that weak adsorptive forces are effective on the surface of activated carbon. Values of *n* > 1 represent a favorable adsorption condition, suitable for highly heterogeneous surfaces. In this study, the values found for *n* were between 1.6 and 1.9 which prove that the adsorption is favorable and process could be physical in nature (Behnamfard & Salarirad 2009; Yan et al. 2008).

## Conclusions

The results reported in this study show that activated carbons ML can be envisaged as alternative adsorbents for acetaminophen removal from SGF. ML did not differ substantially from commercial standards NB and NE. The CO_2_ isotherms indicate that ML is a microporous material with a texture similar to the NE patterns. From the comparison of three activated carbons with different nature, it is clear that both the microporous structure and the surface chemistry have a critical role in defining the adsorption capacities.

It was determined that the adsorption process of acetaminophen on activated carbon in SGF is spontaneous (ΔG <0). As seen in most cases. the models fit very well with the data analyzed, depending on the type of activated carbon. The adsorption of acetaminophen may occur in specific sites and also in the basal region. Although it is well established that the oxidation process is performed by the oxygen surface groups, previous results show that not only the amount but also the nature of these oxygen groups and distribution becomes crucial. The estimated calculation of the area of the section the acetaminophen molecule transversal demonstrates that affirmation.
